# Lung abscess by *Fusobacterium nucleatum* and *Streptococcus* spp. co-infection by mNGS: A case series

**DOI:** 10.1515/biol-2022-0651

**Published:** 2023-07-17

**Authors:** Na Wang, Zhichao Gao, Shuai He, Mengzhen Han, Wenjie Han, Xiaolin Liu, Hui Cao, Mingxi Jing, Tao Sun, Junnan Xu

**Affiliations:** Department of Breast Medicine, Cancer Hospital of China Medical University, Liaoning Cancer Hospital, Shenyang, 110042, China; Department of Pharmacology, Cancer Hospital of China Medical University, Liaoning Cancer Hospital, Shenyang, 110042, China; Department of Breast Medicine, Cancer Hospital of Dalian University of Technology, Liaoning Cancer Hospital, Shenyang, 110042, China; Department of Imaging, Cancer Hospital of Dalian University of Technology, Liaoning Cancer Hospital, Shenyang, 110042, China; Liaoning Kanghui Biotechnology Co., Ltd, Zhihuier Street, Hunnan District, Shenyang, 110001, China; Department of Medical Oncology, Cancer Hospital of Dalian University of Technology, Liaoning Cancer Hospital and Institute, No. 44 Xiaoheyan Road, Dadong District, Shenyang, Liaoning 110042, P. R. China

**Keywords:** co-infection, *Fusobacterium nucleatum*, lung abscess, shotgun metagenome next-generation sequencing, *Streptococcus* spp.

## Abstract

A lung abscess is a necrotizing infection caused by microbiomes that lead to the loss of healthy lung tissue. The routine culture is a waste of time and yields false-negative results, and clinicians could only choose empiric therapy or use broad‐spectrum antibiotics, which could significantly contribute to the problem of resistance or aggravate the condition. We report three patients with a routine-culture-negative lung abscess. The presenting symptoms included fever, cough, dyspnea, and chest pain, and a computed tomography scan revealed a lesion in the lungs. The bronchoalveolar lavage fluid and pleural fluid were tested for pathogens using metagenome next-generation sequencing (mNGS), and the results revealed *Fusobacterium nucleatum* and *Streptococcus* spp. (*S. constellatus*, *S. intermedius*) as the most represented microbial pathogens. Our data demonstrated that mNGS could be a promising alternative diagnostic tool for pathogen detection, and the pathogen lists indicate that it will be important to focus on the *Streptococcus* genus rather than the dominant *Streptococcus* spp. in terms of co-infection of pathogen determined by shotgun mNGS.

## Graphical abstract

The aim of this article was to employ shotgun mNGS for pathogen diagnosis in three patients with lung abscesses. The pleural fluid or bronchoalveolar lavage fluid from patients was sent to traditional culture and the shotgun mNGS test. Laboratory culture tested negative and shotgun mNGS analysis revealed a co-infection caused by *Fusobacterium nucleatum* and *Streptococcus* spp. After standard treatment of co-infection, patients gradually improved and were discharged. This figure is drawn by Figdraw.

**Figure:**
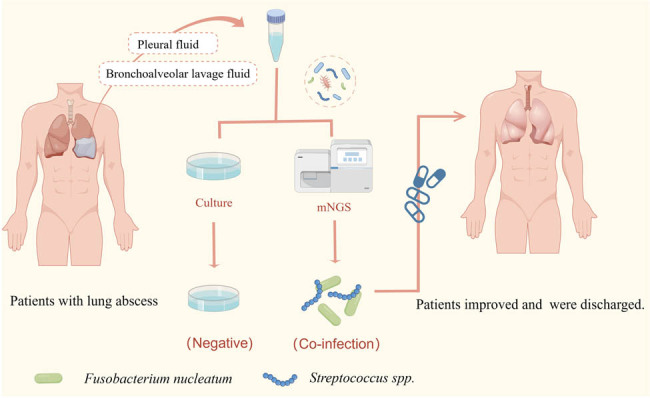

## 1 Introduction

Lung abscess is defined as a microbial infection in lung parenchyma, and typically exhibits a cavity (more than 2 cm) containing necrotic debris or fluid [1]. It continues to be a fatal disease, despite significant progress made in percutaneous drainage and antibiotic therapy. Based on the species of pathogenic microorganisms, lung abscesses could be divided into mono-microbial and polymicrobial infections, which have major differences in antibiotic therapy and prognosis [2]. Mono-microbial lung abscess is caused by *Streptococci*, *Staphylococcus aureus*, *Acinetobacter baumannii*, *Klebsiella pneumoniae*, *Pseudomonas aeruginosa,* and so on [3]; while polymicrobial infection is common and is up to 72.2% [4], containing multiple anaerobic bacteria. *Fusobacterium nucleatum (Fn)* and *Bacteroides* spp. are predominant among all anaerobes. For known microbial pathogens, different pathogens may be treated with different antibiotics. For example, vancomycin or linezolid are preferred for methicillin-resistant *S. aureus* infection, while for *Legionella micdadei* the choice is the macrolide antibiotic. Metronidazole, as a single therapy, does not appear to be particularly useful due to polymicrobial flora [3]. There is evidence that adhesion and invasion protein can influence bacterial co-aggregation, resulting in increased chances of co-infection [4]. Early diagnosis of pathogens contributes to facilitating prompt antibiotic treatment, while virulence gene analysis contributes to the understanding of the precise mechanism of co-infection.

Currently, clinical microbial pathogen detection is still dominated by traditional methods such as culture, staining microscopy, and PCR, which have limitations such as long culture cycles and low sensitivity. For example, the identification of mycobacterium strains takes up to 30 days, while some anaerobic bacteria and viruses have extremely strict requirements on culture conditions, and even cannot be cultured. Although PCR-based molecular diagnostic technology has solved the above problems of pathogen identification, it is difficult to solve the detection problem of unknown microorganisms because primers cannot be designed for nucleic acid sequences of unknown microorganisms [5]. Metagenome next-generation sequencing (mNGS) has the ability to overcome the limitations of these traditional microbial pathogen detection methods and can be directly sequenced and identified without relying on known nucleic acid sequences [6]. As an unbiased pathogen detection method, mNGS can be used in many diseases caused by infection, including central nervous system infections, bloodstream infections, and ocular infections [7–9].

The presence of commensal oral flora organisms, which in some cases may also be pathogens, further complicate pathogen diagnosis in respiratory infections. One of the most striking advantages of mNGS is the ability to distinguish infection from colonization. Furthermore, the administration of broad-spectrum antimicrobial agents may render it difficult to routine culture microorganisms, impairing pathogen diagnosis. mNGS is also capable of overcoming the limitations of this traditional culture. Another advantage of mNGS is that it enables the unbiased and rapid detection of high numbers of pathogens, ranging from bacteria to fungi, viruses, and parasites, addressing the limitation of missed detections. Clinical guidelines and expert consensus have also confirmed that mNGS has greatly enhanced our ability to diagnose, interrogate, and track infectious diseases, as well as these techniques allow researchers to investigate variation in the microbial community structure in a culture-independent manner [10,11]. Lung abscess co-infection is always culture-negative and the possibility of missed detection is also present in clinical setting, which mainly relies on empiric therapy. Herein, we report three cases of lung abscesses caused by *Fn* combined with *Streptococcus* (including *S. constellatus* and *S. intermedius)* using shotgun mNGS, as well as detecting the presence of virulence genes FadA and RadD, which may be useful to search co-infectious mechanism.

## 2 Patients and methods

### 2.1 Patients

This study enrolled three patients with lung abscesses of *Fn* and *Streptococcus* co-infection who were admitted to Liaoning Cancer Hospital between April 2021 and June 2021. The criteria for patient inclusion were the following: (1) the patient presented with symptoms including fever, cough, dyspnea, and chest pain; (2) imaging examination showed that the lungs appear as cavitary lesions with infiltrates; (3) *Fn* and *Streptococcus* were detected by mNGS with relative abundance in the top 2, and no other pathogenic bacteria that could cause lung abscesses such as *Klebsiella pneumonia*, *S. aureus*, and *Mycobacterium* were detected in the top 10 or their abundance was less than 1%; and (4) the subjects gave their permission for this study within the informed consent form.


**Informed consent:** Informed consent was obtained from all individuals included in this study.
**Ethical approval:** The research related to human use complied with all the relevant national regulations, institutional policies, and is in accordance with the tenets of the Helsinki Declaration, and has been approved by the Ethics Committee of Liaoning Cancer Hospital (20201135K).

### 2.2 DNA extraction, library preparation, and sequencing

Total DNA from BALF and pleural fluid were extracted using the nucleic acid extracted kit (51304, QIAGEN, Germany) and purified using DNA Purification Magnetic Beads (Vazyme, China). DNA libraries were then constructed using the DNA Library Prep Kit (NDM617, Vazyme, China) and sequenced with a 100 bp single-end protocol on an MGISEQ-2000RS sequencing platform.

### 2.3 Quality control of reads and removal of human reads

Multiple open-source and private software packages were employed to process the raw sequences. Accordingly, sequences were deduplicated, quality trimmed, and adapter-removed using clumpify from the BBTools suite and fastp. The sequences that were mapped to the human reference genome GRCh38 were eliminated using Bowtie2 with options p 16. A secondary analysis of sequences was undertaken using BMTagger software in order to further exclude human host reads. A FASTQ file was then generated from reads, which did not align using Samtools (view -b -f 4, -F 256).

### 2.4 Taxonomic classification and verification

The FASTQ file was analyzed using two different classifiers: KMA (v 1.3.6) and Blastn (v 2.10.1). The two software packages were chosen to ensure the objectivity and accuracy of our research. KMA was run against the laboratory-developed microbial databases that included 16,959 bacteria, 314 fungi, 9,010 viruses, 173 parasites, 208 mycobacteria, and 162 mycoplasma/chlamydia to determine pathogens and their relative abundance. Blastn was run using options -max_target_seqs 5, -num_threads 10, -outfmt 6 and -evalue 1 × 10^−10^ on the NCBI-nt database. Principal component analysis (PCA) biplots were generated from the pathogen data using the PCA online analysis website (http://www.ehbio.com/Cloud_Platform/front/).

### 2.5 Prediction of virulence genes for *Fn*

The completed reference sequences of *Fn* were downloaded from the NCBI database to identify virulence genes FadA and RadD. The coverage and depth of *Fn* and its FadA and RadD genes in the three patients were generated and the formulae are as follows: Coverage = (# area covered by mapped reads)/(# area of reference) and Depth = (# of bases mapping to the locus)/(size of locus). *Fn* reads were assembled by SPAdes (v3.15.2) with default parameters, except for kmer sizes (kmer = 35). Scaffolds of short (<500 bp) were filtered out. The quality of genome assembly was assessed using the QUAST version 5.0.2 with default parameters. The known FadA signal peptide protein sequences (MKKFLLLAVLAVSASAFA) as queries to search gene models of *Fn* were determined using BLASTP (v2.10.1), with an *e*-value threshold of 1 × 10^−5^.

## 3 Clinical presentation

### 3.1 Case 1

**Figure 1:**
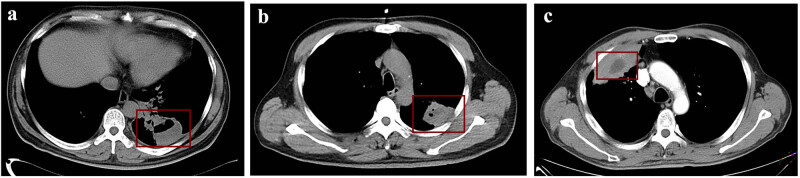
(a) Chest CT revealed cavitary lesion (56 mm × 41 mm) in the lower lobe of the left lung in case 1. (b) Chest-enhanced CT of case 2 showing a mass in the upper lobe of the left lung (about 45 × 34 mm). (c) A ches- enhanced CT scan revealed a soft tissue mass shadow in the upper lobe of the left lung in case 3.

**Figure 2:**
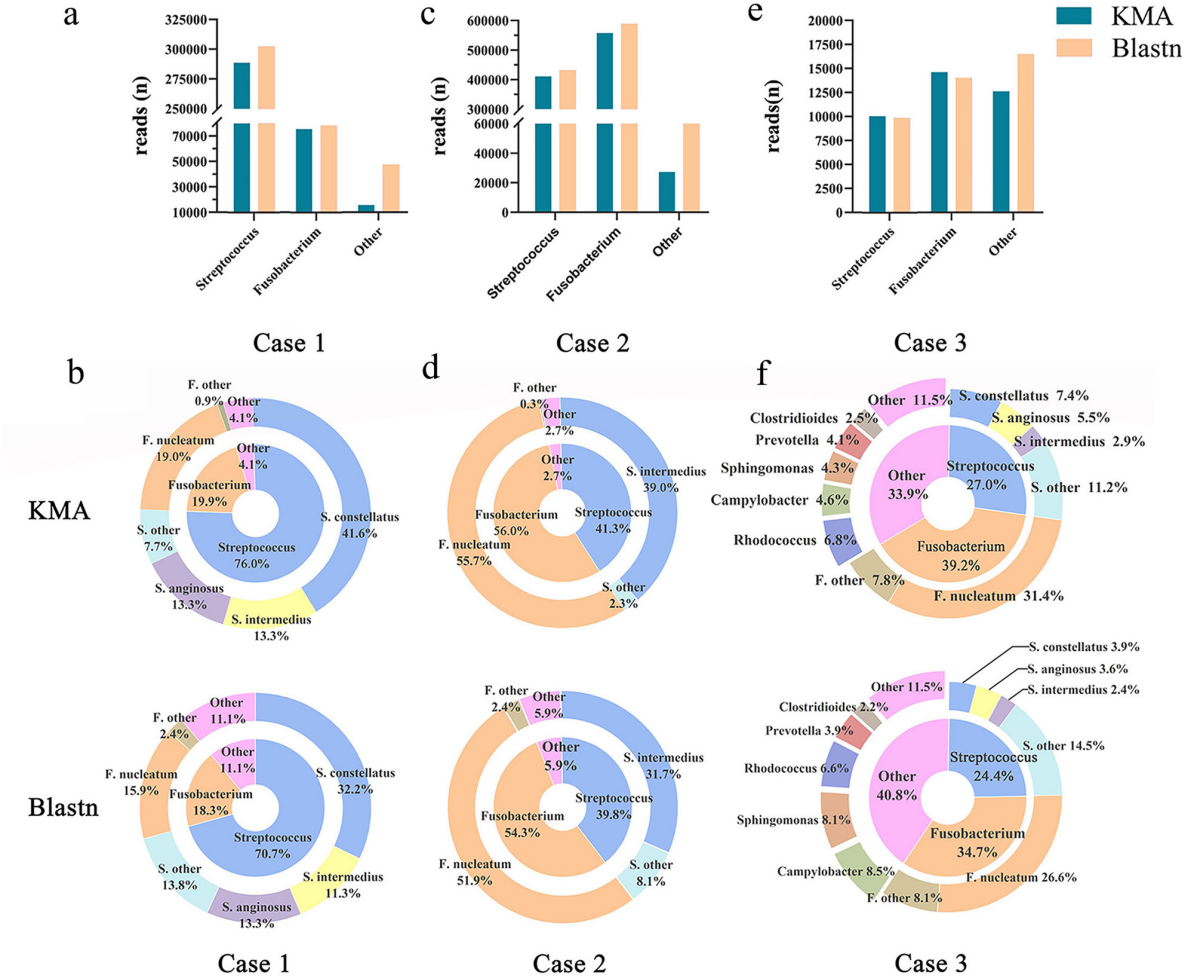
Read numbers and relative abundance of pathogens in three patients using two different taxonomic software. (a) The read numbers of pathogens at the genus level. (b) Taxonomic composition and relative abundance of pathogens at the species level.

A 76-year-old female was transferred to us with chest distress, chest pain for 1 month, severe cough, and expectoration to last 3 days. The patient was in poor physical condition and had a past medical history of hypertension and diabetes for over 15 years and pulmonary fibrosis for 3 years. The initial analysis revealed the following: white blood cell (WBC) count of 8.0 × 10^9^/L, neutrophil proportion of 71.0%, lymphocyte proportion of 23.4%, C-reactive protein (CRP) of 16.4 mg/L, and pro-calcitonin (PCT) of 0.05 μg/L. Thoracic computed tomography (CT) scanning indicated a 56 mm × 41 mm cavitary lesion in the lower lobe of the left lung, and spots, patches, and solid shadows surrounded the mass. A left lower lobe lesion with soft tissue density and pleural effusion was present (Figure 1a). The patient was diagnosed with a pulmonary abscess, treated with intravenous mezlocillin and ornidazole for 4 days, and the disease progressed. The patient underwent CT-guided percutaneous drainage and approximately 30 mL of thick pus was removed; the pleural fluid was sent for culture and for the shotgun mNGS test. The laboratory culture results were negative. Shotgun mNGS analysis revealed that *Fn* has 72,426 reads and the abundance was 19.0%; the 288,845 reads corresponded to the *Streptococcus* genus, in which *S. constellatus* was up to 54.8% (Figure 2a and 2b). *Fn* and *S. constellatus* were considered potential pathogens. Virulence genes FadA and RadD were detected because the former is the best-characterized virulence gene of *Fn* and the latter possesses the ability to bond with the *Streptococcus* spp. The coverage of FadA was only 7.2% and no signal peptides were detected, while the RadD gene coverage was 66.5%. *Streptococcus* spp. was excluded from virulence genes analysis because of high homology results in multiple mapping. The symptoms were gradually relieved when the patient was switched to piperacillin-tazobactam; the patient was discharged after 10 days.

### 3.2 Case 2

A 40-year-old female experienced pain in the right sternocostal part for 4 days. Chest enhanced CT showed a mass in the upper lobe of the left lung, about 45 mm × 34 mm in size. After enhanced CT scan, no enhanced areas was visible inside, and aerated bronchus sign inside the lesion. The lesion spans the interlobular fissure to the dorsal segment of the left lower lobe of the lung, and no obvious mass was present on the chest wall (Figure 1b). The laboratory test showed a WBC count of 11.4 × 10^9^/L, 74.2% neutrophils, 17.2% lymphocytes, 127.2 mg/L CRP, and 0.07 μg/L PCT. She was treated with levofloxacin. After 2 days, she developed an intermittent high fever, which was up to 39.5℃. The sputum culture grew a normal oral microbiome, and the analysis of BALF with shotgun mNGS reported *Fn* (554,856 reads, abundance: 55.7%) and *Streptococcus* genus (410,708 reads, abundance: 41.3%). *S. intermedius* was the most frequent species, which was up to 94.4% (Figure 2c and 2d). Virulence genes FadA of *Fn* had 50.8% coverage, with complete signal peptides (coverage 100%) and RadD coverage was 59.7%. The antibiotic treatment was changed to biapenem. Her chest discomfort diminished and her stomach upset symptoms improved. After 4 days, the blood test showed a WBC count of 6.7 × 10^9^/L, 45.3% neutrophils, 43.5% lymphocytes, 0.8 mg/L CRP, and 0.04 μg/L PCT. The patient was symptomatically better and discharged.

### 3.3 Case 3

A 59-year-old male presented with sustained fever and chest pain for 3 days. The highest body temperature was up to 39℃. A chest-enhanced CT scan revealed a soft tissue mass shadow in the upper lobe of the left lung, a large consolidation shadow in the distal part, and no small nodular shadows were seen around. A few solid shadows in the lower lobes of both lungs were seen (Figure 1c). The blood test showed a WBC count of 9.9 × 10^9^/L, 78.9% neutrophils, 12.7% lymphocytes, 164.0 mg/L CRP, and 0.11 μg/L PCT. He was treated with etimicin for 2 days, but the fever continued. The patient underwent ultrasound-guided percutaneous drainage, and 75 mL of pleural fluid was removed and sent for pathogen testing. The routine culture had no bacteria growth. Shotgun mNGS analysis revealed *Fs* and *Streptococcus* genus accounting for 66.2% of the total abundance, and a low amount of diversified microbiomes were detected such as *Rhodococcus* (6.8%) and *Campylobacter* (4.6%) (Figure 2e and 2f). It was identified that the patient was co-infected with *Fn* (11,716 reads, abundance: 31.4%) and *Streptococcus* genus (10,066 reads, abundance: 27.0%). The patient was excluded from virulence gene analyses because the coverage of *Fn* was only 35.1% and poor assembly quality may lead to misjudgment. The patient was switched to meropenem for 8 days, and the patient’s body temperature returned to normal gradually and the pain dissipated. The CT scan showed radiological improvement and the patient was discharged. In fact, the chest-enhanced CT scan revealed a soft tissue mass shadow in the upper lobe of the left lung. The clinician suspected the possibility of a tumor and recommended surgery but the patient did not accept it for financial reasons. The basic clinical information and mNGS results in the three patients with lung abscesses are summarized in Table 1.

**Table 1:** Basic clinical information and mNGS results in three patients with lung abscess

Case	Age, gender	Potential risk factor	Sample type	Medication history	mNGS result		Treatment	Outcomes
					*Fn* and *Streptococcus* (%)	*Fn* and dominant *Streptococcus* (%)		
1	76, F	Diabetes	Pleural fluid	Mezlocillin, ornidazole	95.0	60.6	Piperacillin-tazobactam + percutaneous drainage	Good evolution (10 days)
2	40, F	None	BALF	Levofloxacin	97.0	94.7	Biapenem	Good evolution (4 days)
3	59, M	None	Pleural fluid	Etimicin	58.4	38.8	Meropenem + percutaneous drainage	Good evolution (10 days)

*Fn*: Fusobacterium nucleatum, BALF: bronchoalveolar lavage fluid.

The patients were followed up by phone call consultation every 2 weeks for a total of 3 months. Cases 1 and 2 showed good tolerability and improved completely without recurrence, and no severe adverse reactions or unanticipated events have been reported. Case 3 was treated with anti-tumor therapy in a higher-grade hospital and additional clinical information is not available.

**Figure 3:**
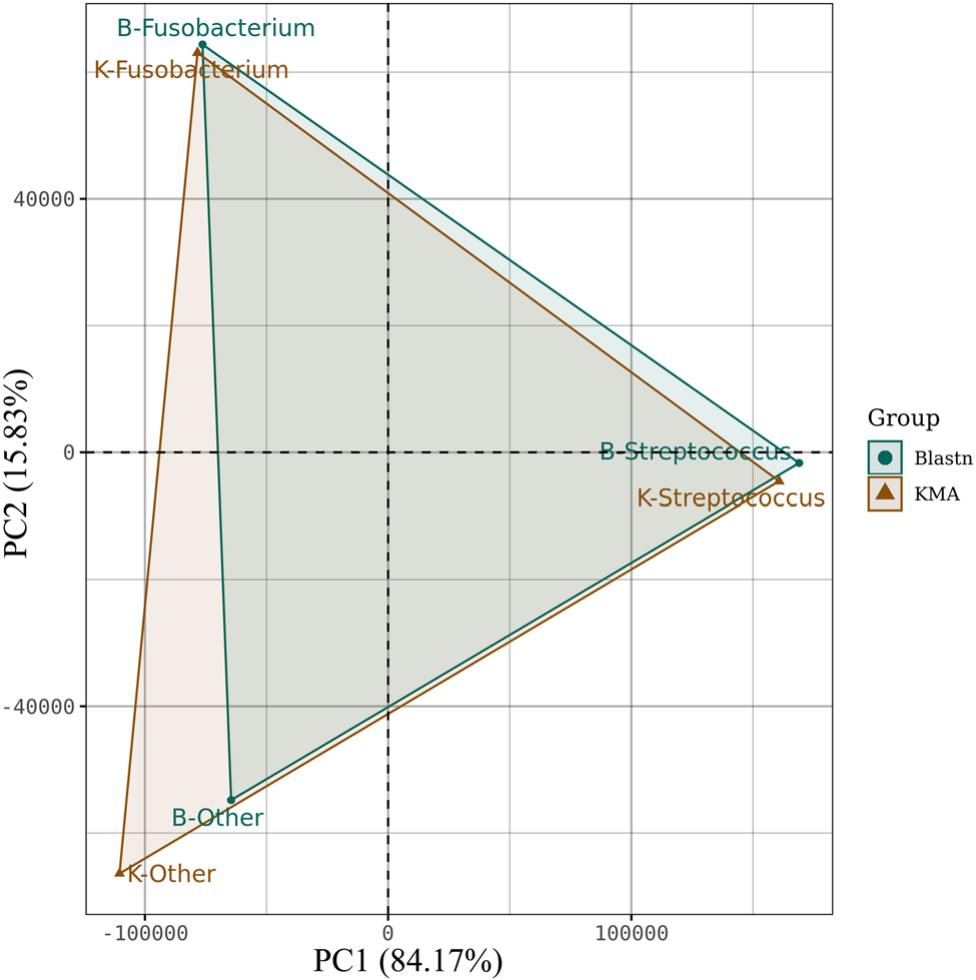
PCA of the datasets in the *Fusobacterium* and *Streptococcus* genus using KMA and Blastn. The green circles and red-brown triangles represent the Blastn and KMA taxonomic methods, respectively. The PCA results for the two taxonomic methods showed similar results in general, which reflects consistency in classification, despite the different methods used.

The standard mNGS protocol is briefly described as follows. It involves a series of DNA extraction, library preparation, sequencing, and data analysis. Total DNA from BALF and pleural fluid were extracted using the nucleic acid extracted kit (51304, QIAGEN, Germany) and purified using DNA Purification Magnetic Beads (Vazyme, China). DNA libraries were then constructed using the DNA Library Prep Kit (NDM617, Vazyme, China) and sequenced with a 100 bp single-end protocol on an MGISEQ-2000RS sequencing platform. Multiple open-source and private software packages were employed to process the raw sequences. Accordingly, sequences were deduplicated, quality trimmed, and adapter-removed using clumpify from the BBTools suite and fastp. The sequences that were mapped to the human reference genomes GRCh38 were eliminated using Bowtie2 with options -p 16. A secondary analysis of sequences was carried out using BMTagger software in order to further exclude human host reads. A FASTQ file was then generated from reads, which did not align using Samtools (view -b -f 4, -F 256). After removing the human sequences, the remaining reads were analyzed using two different classifiers KMA and Blastn to ensure the objectivity and accuracy of our research. KMA was run against a laboratory-developed microbial databases that included 16,959 bacteria, 314 fungi, 9010 viruses, 173 parasites, 208 mycobacteria, and 162 mycoplasma/chlamydia. The parameters of KMA are set as -sam 4, -t_db, -1t1, -t 16, -mem_mode -cge -ef -a. Blastn was run using options -max_target_seqs 5, -num_threads 10, -outfmt 6 and -evalue 1 × 10^−10^ on the NCBI-nt database. To estimate the microbiome community structure differences in different taxonomic classification methods, a PCA was performed and reflects *Fs* and *Streptococcus* consistency in classification, despite particular deviations in relative abundance (Figure 3). The Blastn confirmed the reliability of the analysis by KMA results, so subsequent analysis of the virulence genes was dominated by the KMA result.

## 4 Discussion

Lung abscess is a severe infection and has a high prevalence worldwide. Early microbial pathogen diagnosis and prompt treatment are crucial for preventing potential fatalities and improving health outcomes. In our study, the most common symptoms in three patients with lung abscesses were fever (66.7%), chest distress, or chest pain (100.0%). These match with those reported in the literature [12]. In addition, the pathogenic microorganisms of lung abscesses have been described in a wide genus species range. For example, Adlakha and Muppala reported a patient with a lung abscess caused by *Propionibacte*rium acnes [13], whereas Hayashi et al. reported a secondary pulmonary abscess caused by *Nocardia asiatica* in a 78-year-old Japanese man [14]. The clinical symptoms and signs of lung abscesses caused by different pathogens are very similar and are often difficult to identify [12]. Therefore, it is essential to identify the pathogen as soon as possible to choose and optimize antibiotic therapy. However, some anaerobic bacteria or viruses have extremely strict requirements on culture conditions, which will result in false-negative results. A 14-year retrospective cohort study conducted by Fernando et al. showed that the positive rate of conventional blood culture in patients with lung abscesses was only 27.9% [15]. Although interventional methods have increased the detection rate of pathogens responsible for lung abscesses, it is still only 60% [16]. Physicians could only prescribe empirical treatment with broad-spectrum antibiotics if cultures are not available, which could lead to resistance or therapy failure. Herein, we employ shotgun mNGS to diagnose the pathogen of three patients with lung abscesses whose conventional pathogen cultures were negative and identified the co-infection by *Fn* and *Streptococcus* spp*. Fn* and *Streptococcus* are difficult to culture in the clinic, which makes the diagnosis of lung abscess pathogen at an early time point an intractable problem. Shotgun mNGS, a revolutionized tool in assisting clinical pathogen diagnosis, plays a unique role in identifying novel and rare pathogens such as *Pneumocystis jiroveci* and *Chlamydia psittaci* [17,18].

A range of similarities exists in congeneric species, that is, multiple mappings may occur in different species. Our study revealed that the *Streptococcus* spp. co-infection with *Fn is* not confined to the same species (*S. constellatus* in cases 1 and 3, *S. intermedius* in case 2). The literature review also suggested that various *Streptococcus* species (*S. anginosus, S. pneumoniae, S. viridans*) could also cause lung abscesses [19,20]. Because of the sequence similarity, the sequences of *Streptococcus* are prone to multiple mapping to more than one species rather than fully matching with a dominant species, especially in case 3. Cases 1 and 3 gradually improved after standard treatment of co-infection and percutaneous drainage intervention, and there are certain risks if pathogens are ignored relying solely on the dominant *Streptococcus* spp. This result gives us a good inspiration: in terms of pathogen determination, the *Streptococcus* genus should be focused on rather than the dominant *Streptococcus* spp. to avoid neglecting pathogens.

A previous fundamental study performed in mice indicated that abscesses caused by the combination of *Fn* and *Streptococcus* spp. tended to be larger than those caused by mono-bacterial infections. The metabolites substantially of *Fn* promote *Streptococcus* spp. growth directly and attenuate the cell-killing abilities of human polymorphonuclear leukocytes on *Streptococcus* spp. [21,22]. In addition, the virulence factor is integral to bacterial colonization and pathogenesis. For example, hypervirulent *K. pneumoniae* carries K1 and K2 capsular serotypes, rmpA, rmp2, and magA genes, resulting in disseminated community-acquired severe infection in immunocompetent hosts, and is more aggressive and metastatic [23]. The virulence factor of *Fn* has a particular emphasis on its adherence and invasion. The protein expressions of FadA and RadD are essential mechanisms for colonization, co-aggregation, and induction of host responses [24,25]. FadA could bind to cadherins, causing the cadherin to migrate from the cell–cell junction to intracellular compartments, increasing the endothelial permeability, and allowing the bacteria to cross the endothelial barrier through loosened junctions [25]. RadD is responsible for arginine-inhibitable adherence in *Fn,* and Kaplan et al. suggested that gene inactivation mutations in RadD demonstrated decreased co-aggregation with *Streptococcus* spp. [26]. Both patients of cases 1 and 2 developed a detectable RadD gene, which also provides evidence to favor the co-infection of *Fn* and *Streptococcus* spp. In terms of antibiotic therapy, *Fn* produced a potent β-lactamase, conferring marked resistance to β-lactam antibiotics such as penicillin [27]. Thus, β-lactam/β-lactamase inhibitor combination agents should be the first choice for co-infection of *Fn* with *Streptococcus.*

The investigation also faces some limitations. The major limitation is that experimental verification was lacking. All patients suffered from negative culture results, and it was not possible to re-sample for PCR and agarose gel verification from patients due to ethical and moral restrictions, leading to the extent of accuracy on pathogenic microorganisms and virulence genes being difficult to assess. Additionally, we were unable to take samples again after treatment for the identification of microbial pathogens because the patients had recovered and were discharged. Another limitation is the sample size; more cases will need to be investigated to verify our findings and to clarify their significance in pathogen diagnosis in subsequent studies.

To the best of our knowledge, this is the first reported study employing shotgun mNGS to diagnose co-infection with *Fn* and *Streptococcus* spp. for lung abscess and tackling the thorny problem of confirming difficult-to-culture pathogens in the clinic. We further analyzed virulence-associated genes for *Fn* in the hope of providing references for future studies on the mechanisms of co-infection with *Fn* and *Streptococcus* spp., as well as new ideas for the choice of antimicrobial agents. Shotgun mNGS is a revolutionary technology that has disrupted traditional clinical diagnostics in terms of time-saving and identification of multiple organisms. Based on this technology, we could further carry out fundamental experimental studies to define inter-bacterial interaction and its relationship with diseases, exploiting novel targets for drug therapy.

## Supplementary Material

Supplementary material
