# Supplementary material for: Lung abscess by Fusobacterium nucleatum and Streptococcus spp. co-infection by mNGS: A case series
Source: Open Life Sci. 2023 Jul 17;18(1):20220651. doi: 10.1515/biol-2022-0651 (PMC10358748; doi:10.1515/biol-2022-0651)
Supplement: Supplementary material [file biol-2022-0651-sm.pdf]

Supplementary material

S1 Patients

This study enrolled 3 patients with lung abscess of *Fn* and Streptococcus co-infection who were admitted to Liaoning Cancer Hospital between April 2021 and June 2021. The criteria for patient inclusion were: (1) The patient presented with symptoms including fever, cough, dyspnea, chest pain; (2) Imaging examination showed the lungs appear as cavitary lesions with infiltrates. (3) *Fn* and Streptococcus were detected by mNGS with relative abundance in the top 2 and no other pathogenic bacteria that could cause lung abscess such as Klebsiella pneumonia, Staphylococcus aureus, Mycobacterium were detected in the top 10 or their abundance is less than 1%; (4) The subjects give their permission for this study within the informed consent form.

S2 DNA extraction, library preparation, and sequencing

Total DNA from BALF and pleural fluid were extracted using nucleic acid extracted kit (51304, QIAGEN, Germany) and purified using DNA Purification Magnetic Beads (Vazyme, China). DNA libraries were then constructed using DNA Library Prep Kit (NDM617, Vazyme, China) and sequenced with a 100-bp single-end protocol on a MGISEQ-2000RS sequencing platform.

S3 Reads quality control and removal of human reads

Multiple open-source and private software packages were employed to process the raw sequences. Accordingly, sequences were deduplicated, quality trimming and adapter removal using clumpify from the BBTools suite and fastp. The sequences that mapping to the human reference genomes GRCh38 were eliminated using Bowtie2 with options -p 16. A secondary analysis of sequences was undertaken using BMTagger software in order to further exclude human host reads. A FASTQ file was then generated from reads which did not align using Samtools (view -b -f 4, -F 256).

S4 Taxonomic classification and verification

The FASTQ file were analysis using two different classifiers: KMA (v 1.3.6) and Blastn (v 2.10.1). The two softwares were chosen to ensure the objectivity and accuracy of our research. KMA was run against a labortary-developed microbial databases that including 16959 bacteria, 314 fungi, 9010 viruses, 173 parasites, 208 mycobacteria and 162 mycoplasma/chlamydia to determine pathogens and its relative abundance. Blastn was run using options -max_target_seqs 5, -num_threads 10, -outfmt 6 and -evalue 1e-10 on the NCBI-nt database. Principal components analysis (PCA) biplots were generated from the pathogen data using the PCA online analysis website (http://www.ehbio.com/Cloud_Platform/front/).

S5 Virulence genes prediction for *Fn*

The completed reference sequences of Fn were downloaded from the NCBI database to identity virulence genes FadA and RadD. Coverage and depth of Fn and its FadA and RadD genes in 3 patients were generated and the formula are as follows: Coverage = (# area covered by mapped reads) / (# area of reference). Depth = (# of bases mapping to the locus) / (size of locus). Fn reads were assembled by SPAdes (v3.15.2) with default parameters, except for kmer sizes (kmer = 35). Scaffold of short (<500bp) were filtered out. Assessing genome assembly quality using the QUAST version 5.0.2 with default parameters. The known FadA signal peptide protein sequences (MKKFLLLAVLAVSASAFA) as queries to search gene models of Fn using BLASTP (v2.10.1), with an *e*-value threshold of 1×10^{-5}.

Tables S1 and S2

Table S1: The number of reads at different steps of data analysis

Case	Total Reads (M)	HQ reads (M)	Nohuman reads (M)	Mapped reads (K)		*Streptococcus* reads (n)		*Fusobacterium* reads (n)	
				KMA	Blastn	KMA	Blastn	KMA	Blastn
1	56.2	53.4 (95.0%)	1.9 (3.5%)	385.6 (20.3%)	443.9 (23.3%)	288,845 (76.0%)	302,535 (70.7%)	75,678 (19.9%)	78,237 (18.3%)
2	40.9	40.9 (99.9%)	3.8 (9.3%)	977.9 (25.2%)	1,085.3 (27.9%)	410,708 (41.3%)	431,988 (39.8%)	557,512 (56.0%)	589,180 (54.3%)
3	21.2	20.1 (94.8%)	0.2 (0.9%)	37.3 (18.1%)	40.4 (19.5%)	10,066 (27.0%)	9,868 (24.4%)	14,620 (39.2%)	14,029 (34.7%)

Table S2: De novo assembly quality of Fusobacterium nucleatum and the coverage and depth of virulence genes FadA and RadD

No.	*Fn* reads (M)	Coverage (%)	Depth (×)	Assembly indicators				FadA (387 bp)			RadD	
				(3526 bp)								
				Total length (k)	Number contigs	N50 (k)	GC (%)	Signal peptide coverage (%)	Coverage (%)	Depth (×)	Coverage (%)	Depth (×)
Ref (GCA_002211645.1)	—	—	—	2322.9	1	—	27.1	100.0	100.0	—	100.0	—
Case 2	55.5	90.1	25.9	1906.4	761	3.7	27.4	100.0	50.8	12.6	59.7	10.9
Case 1	7.2	81.4	3.7	474.5	673	0.7	28.4	0.0	7.2	2.4	66.5	2.7
Case 3	1.2	35.1	1.4	0.5	1	0.5	49.0	—	—	—	—	—

Fn: Fusobacterium nucleatum.
